# Copper-containing glass ceramic with high antimicrobial efficacy

**DOI:** 10.1038/s41467-019-09946-9

**Published:** 2019-04-30

**Authors:** Timothy M. Gross, Joydeep Lahiri, Avantika Golas, Jian Luo, Florence Verrier, Jackie L. Kurzejewski, David E. Baker, Jie Wang, Paul F. Novak, Michael J. Snyder

**Affiliations:** 1 Corning Incorporated, Corning, NY 14831 USA; 2 Argonne National Laboratory, Argonne, IL 60439 USA

**Keywords:** Antimicrobials, Antimicrobial resistance, Policy and public health in microbiology, Nanoparticles

## Abstract

Hospital acquired infections (HAIs) and the emergence of antibiotic resistant strains are major threats to human health. Copper is well known for its high antimicrobial efficacy, including the ability to kill superbugs and the notorious ESKAPE group of pathogens. We sought a material that maintains the antimicrobial efficacy of copper while minimizing the downsides – cost, appearance and metallic properties – that limit application. Here we describe a copper-glass ceramic powder as an additive for antimicrobial surfaces; its mechanism is based on the controlled release of copper (I) ions (Cu^1+^) from cuprite nanocrystals that form in situ in the water labile phase of the biphasic glass ceramic. Latex paints containing copper-glass ceramic powder exhibit ≥99.9% reduction in *S. aureus*, *P. aeruginosa, K. aerogenes* and *E. Coli* colony counts when evaluated by the US EPA test method for efficacy of copper-alloy surfaces as sanitizer, approaching that of benchmark metallic copper.

## Abstract (web summary)

Copper is well known for its high antimicrobial efficacy; however, cost, appearance and metallic properties limit application. Here the authors describe a stable, water dispersible copper-glass ceramic powder with long-term antimicrobial activity as an additive for antimicrobial surfaces.

## Introduction

Nosocomial infections and the emergence of antibiotic-resistant strains pose significant clinical and economic challenges^1, 2^. While good hygiene practices are the bedrock for infection control, emerging evidence suggests that continuously killing antimicrobial surfaces based on metallic copper can reduce bioburden and lower the risk of infection^3^. Copper’s multiple mechanisms of action, which includes the ability to destroy genomic and plasmid DNA^4^, explain its longstanding antimicrobial efficacy against pathogens such as antibiotic-resistant “superbugs”^5, 6^. Proposed mechanisms for copper-mediated cellular damage and toxicity include direct cell membrane damage, the generation of reactive hydroxyl radicals through Fenton-type reactions, and entry of copper ions into cells through ligand interactions causing disruption of RNA and DNA function^5–7^. While the precise mechanism is unclear, Cu^1+^ ions are considerably more toxic to bacteria than Cu^2+^ ions under test conditions that mimic microbial contamination on solid surfaces^8–10^.

There has been a significant focus on metallic copper as an antimicrobial since the US EPA introduced a new test protocol in 2008 that was exclusively passed by copper surfaces^11, 12^. The US EPA mandated that claims of efficacy against human pathogens could only be obtained for products that pass the standard with the justification that it was a realistic simulation of contamination unlike the traditional test^6, 13^. We wanted to make a copper-containing additive compatible with commonly used surfaces and coatings that demonstrated the efficacy to kill ≥99.9% of bacteria under EPA’s test conditions retaining copper's broad spectrum efficacy and low probability for the development of resistant strains^5, 14^.

We describe an alkali copper aluminoborophosphosilicate glass ceramic material that acts as a sustainable delivery system for Cu^+1^ ions with high antimicrobial efficacy. We use paint coatings as a first-case demonstrative application due to their ubiquity. Paint coatings containing the copper–glass ceramic powder exhibit ≥99.9% reduction in *S. aureus*, *P. aeruginosa*, *K. aerogenes*, and *E. coli* colony counts using the US EPA test method. The copper–glass ceramic is advantaged over existing organic antimicrobial paint additives owing to a favorable toxicological profile and copper’s broad-spectrum efficacy. Unlike air and moisture sensitive cuprous compounds, the copper–glass ceramic is environmentally stable and disperses easily in water. Our work showcases an effective and versatile antimicrobial additive with potential application in coatings and plastics. It also demonstrates rational glass design as an approach for the practical synthesis of functional nanomaterials.

## Results and discussion

### Copper–glass and copper–glass ceramic compositions

**Fig. 1:**
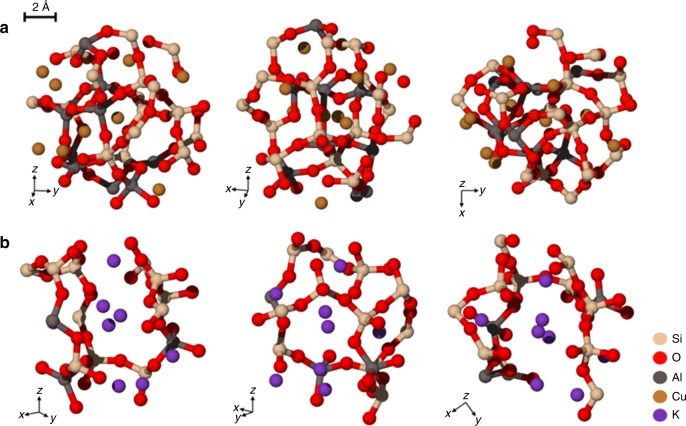
Structures of two model glass compositions by molecular dynamics simulations **a** Model glass with composition of 60SiO_2_−20Al_2_O_3_−20Cu_2_O (in mol%). The model shows a tightly packed glass network structure surrounding small, high-field strength copper (I) ions. **b** Model glass with composition of 60SiO_2_−20Al_2_O_3_−20K_2_O (in mol%). The model reveals a more open glass network structure surrounding larger, lower-field strength K^+^ ions. The high oxygen packing density of glass networks formed around small radius, high ionic field strength modifier ions (as in **a**) reduces the capability for ion exchange with charged water species

We investigated copper-containing glasses because we hypothesized that the excellent barrier properties of glass to gaseous oxygen^15, 16^ would stabilize the environmentally labile Cu^1+^ ions. Copper in aluminosilicate glasses has been described and demonstrated to contain >70% of copper in the Cu^1+^ state^17, 18^. Since monovalent ions within glasses can be extracted via an ion exchange process with H_3_O^+^ ions^19, 20^, this system was a promising starting point for the design of antimicrobial glasses. The initial composition that was melted was batched as 60SiO_2_−20Al_2_O_3_−20CuO (“Methods”). The analyzed composition for this glass (Sample A) is provided in Table 1. Our copper redox analysis using inductively coupled plasma–optical emission spectroscopy (ICP-OES) for total copper and Cr/Cu redox reaction for total Cu^1+^ indicated that the ratio of Cu^1+^ to total copper was 0.86 for the copper–glass. Unlike previous experiments aimed at reducing copper^21^, the melting was not performed under a reducing atmosphere or with batched reducing agents. The reduction of copper from Cu^2+^ in the batched material to Cu^1+^ in the final glass was attributed to the high melting temperature and the nominally reducing conditions attributed to the glass composition. Glass compositions rich in high field strength cations such as Si^4+^ and B^3+^ have a strong reducing effect on multivalent ions incorporated into the glass melt^22^. Unfortunately, the glass, when milled into a powder and mixed into paint, did not show the desired antimicrobial potency. We hypothesized that the small ionic size of Cu^1+^ (*r* = 0.60 Å)^23^ would limit the ion exchange reaction with larger H_3_O^+^ (*r* = 1.4 Å)^24^ as the extraction mechanism. When a glass is cooled from the molten state, the oxygen packing density of the glass network is increased as ionic field strength of modifier ions such as Cu^1+^ increases, resulting in smaller interstitial spaces surrounding the modifiers. Since inter-diffusion of charged species requires charge neutrality^25^, the H_3_O^+^/R^1+^ (R = Li, Na, K, Cu, etc.) ion exchange is effectively stopped if the larger ion cannot enter the site that forms around the smaller, higher field strength ion. Figure 1a, b shows structures of two model glasses with compositions of 60SiO_2_−20Al_2_O_3_−20Cu_2_O and 60SiO_2_−20Al_2_O_3_−20K_2_O obtained through molecular dynamics simulations (“Methods”). The structures demonstrate the steric constraints that limit ion exchange between H_3_O^+^ ions with Cu^1+^ but not with K^+^ (*r* = 1.4 Å)^24^, consistent with experimental observation^26^. Thus, while the initial ternary glass contained significant amounts of Cu^1+^ ions, there was no mechanism to effectively extract these ions from the glass for antimicrobial activity.

**Table 1:** Copper–glass and copper–glass ceramic compositions

Analyzed composition (mol%)	Sample A	Sample B	Sample C	Sample D	Sample E	Sample F
SiO_2_	65.47	68.58	70.53	69.23	66.25	60.12
Al_2_O_3_	22.45	16.20	10.10	0.00	1.96	1.67
CuO	3.02	2.00	2.16	3.43	2.61	3.98
Cu_2_O	9.06	8.31	8.19	8.39	10.06	14.64
K_2_O	0.00	4.91	9.03	9.46	6.21	6.98
P_2_O_5_	0.00	0.00	0.00	0.00	5.26	5.38
B_2_O_3_	0.00	0.00	0.00	9.49	7.63	7.23
Cu^1+^/Total Cu	0.86	0.88	0.86	0.80	0.86	0.88
Crystalline species	None	Tenorite	Tenorite and cuprite	Cuprite	Cuprite	Cuprite
Color	Black	Black	Brown	Yellow	Orange	Orange
Phase separated	No	No	No	No	Yes	Yes
Log Kill (EPA method^7^)	<1	<1	>2	>2	>4	>5

**Fig. 2:**
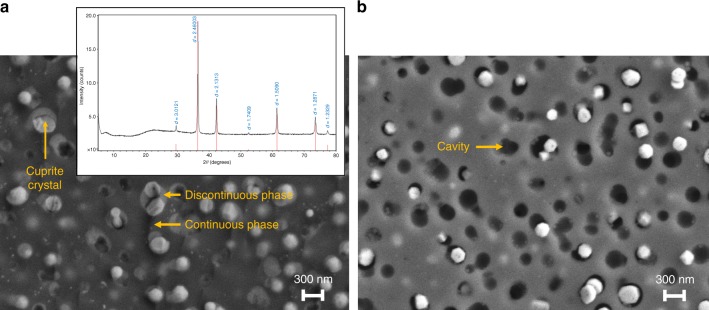
Scanning electron microscope (SEM)  images of copper–glass ceramic cross-section before and after exposure to water **a** Cross-section of the copper–glass ceramic shown prior to water exposure reveals a continuous glassy phase, a discontinuous glassy phase, and a cuprite crystalline phase trapped in the discontinuous phase. Inset is X-ray diffraction (XRD) data collected on copper–glass ceramic powder which shows that the crystalline species present is cuprite. **b** Cross-section of the copper–glass ceramic shows the formation of cavities following water exposure (~120 min) due to dissolution of the discontinuous glassy phase that lead to the release of cuprite crystals into solution

**Fig. 3:**
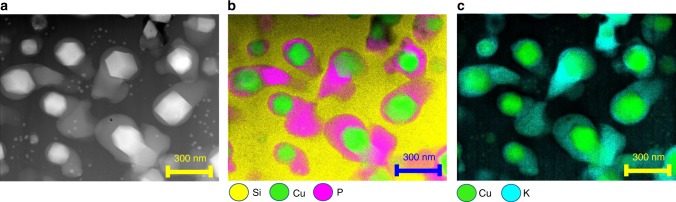
Elemental mapping of chemical species in microstructure of copper–glass ceramic Energy-dispersive X-ray spectroscopy (EDS) using a scanning transmission electron microscope was used for elemental mapping. **a** A microstructural view of the copper–glass ceramic highlighting the three phases present. Some smaller cuprite crystals are also observed in the continuous phase. **b** EDS elemental mapping shows that the high durability, continuous matrix phase is enriched in silicon, the lower durability, discontinuous phase in enriched in phosphorous, and the crystals are composed of copper. **c** EDS elemental mapping also reveals that the lower durability phase is enriched in potassium. Although boron is a major element in the composition, it was not included in the analysis due to the difficulty of measuring light elements via EDS

To overcome the antimicrobial inefficacy of Cu^1+^-containing glasses, we considered a new approach. The concept was to design a glass that phase separates into a highly durable matrix phase and a lower durability second phase that contains the monovalent copper. We reduced the Al_2_O_3_ content in the glass and replaced it with K_2_O, B_2_O_3_, and P_2_O_5_. Al_2_O_3_ suppresses phase separation in most glass systems, while additions of alkali oxide, B_2_O_3_, and P_2_O_5_ have the opposite effect^27^. The results of several iterations (Samples B–F, Table 1) are shown in Table 1. The level of Cu^+^/total copper remains approximately the same (0.8–0.9) across the samples; we believe that the high melting temperature and silica-rich environment common to all the samples drives that ratio. Low levels of potassium inclusion (Samples B) were insufficient to significantly increase the antimicrobial potency; a marginal increase in potency was observed when approximately 10 mol% of Al_2_O_3_ was replaced by K_2_O (Sample C). The formation of cuprite crystals was observed by powder X-ray diffraction (XRD) at this level of substitution. Higher Al_2_O_3_ content stabilizes monovalent cations such as Cu^1+^ in the glassy phase in a charge compensating role, which presumably necessitates lowering Al_2_O_3_ levels significantly for cuprite crystal formation^27^. Further substitution of Al_2_O_3 _by B_2_O_3_ (Sample D) was made to induce phase separation, but this effect was not observed until P_2_O_5_ was also introduced (Sample E). This biphasic glass containing cuprite showed a major improvement in antimicrobial potency relative to the previous samples. An exemplary composition based on sample E that also included a higher level of copper (Sample F) was shown to have >99.999% efficacy under EPA’s test conditions. This material had a distinct orange color. XRD confirmed that the crystalline phase was cuprite (Cu_2_O) for sample E (Fig. 2a inset). Since this composite material contains both amorphous and crystalline regions, it is generally referred to as a glass ceramic. Figure 2a, b are scanning electron microscopic (SEM) images of the material before and after exposure to water, respectively. The lower durability glassy phase regions containing the bright crystalline facets of cuprite crystals (~300 nm; Fig. 2a) are replaced to a large extent by cavities (Fig. 2b), indicating the release of some of the cuprite crystals from the material. Dissolution of the lower durability phase leads to tunneling into the subsurface with separated discontinuous phases becoming connected and providing additional access to cuprite crystals. Figure 3 is a scanning transmission electron microscopic (STEM) image of the material: the darkest region is the continuous glassy phase, the next lighter regions are the discontinuous glassy regions, and the brightest regions are the faceted cuprite crystals. Composition mapping by electron-dispersive spectroscopy (EDS) shows that the continuous glassy matrix phase is composed primarily of silica while the discontinuous phase containing the cuprite crystals is enriched in phosphorus, boron, and potassium. The dissolution of the discontinuous phase was further proven by analysis of the leached ions measured by ICP-MS. High concentrations of potassium, phosphorus, and boron were observed; conversely, Al ions, presumably present in the silica-rich continuous phase, were present at very low concentrations (Supplementary Table 1).

We hypothesize that the discontinuous phase undergoes facile ion exchange between K^+^ and H_3_O^+^ ions followed by the impregnated water molecules participating in hydrolysis reactions that break oxygen-phosphorus bonds. The hydrolysis reaction has been shown to preferentially take place at Q^3^ groups, i.e., phosphorous atoms linked into the network by three bridging oxygen atoms^28^. The breaking of the chains at Q^3^ locations depolymerizes the phosphorous glass network to release the cuprite crystals. The continuous silica-rich network tempers access to the dispersed phosphorus-rich phase deeper in the particles enabling the overall controlled release of copper ions from the material. We cannot rule out some limited leaching of copper ions from cuprite crystals in the durable silica-rich phase.

### Antimicrobial efficacy of copper–glass ceramic particles

**Fig. 4:**
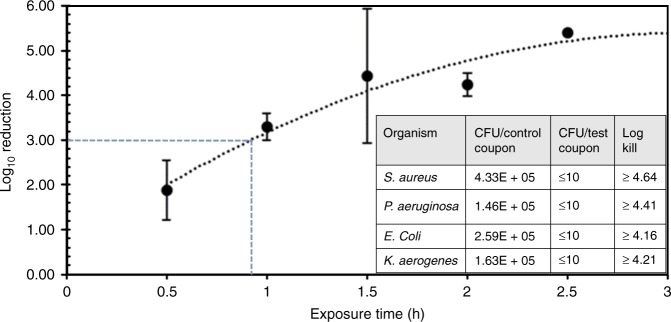
Bacterial reduction kinetics on paint coupons containing copper glass ceramic particles. Plot of *S. aureus* reduction as a function of exposure time shows a steady decrease in *S. aureus* colony counts over 2.5 h, at which point bacterial colonies reduced down to limit of detection. EPA-prescribed 3-log kill (99.9% reduction) was observed in about 1 h. Data and error bars represent mean and standard deviation from two experiments with samples in triplicate. The inset table showcases the high bactericidal efficacy of copper–glass ceramic particles against *S. aureus*, *P. aeruginosa*, *E. coli* and *K. aerogenes.* ≥4-log kill (≥99.99% reduction) was measured against all bacteria following 2-h exposure to test coupons. Bacterial growth on each test and control coupon was measured in triplicate—data shown are geometric mean of values

Copper–glass ceramic particles were air jet-milled with no size classification for reasons of scalability and economics. This gave a particle size *d*_50_ ranging from 2.5 to 5.0 µm. Milled particles were mixed in commercially available decorative paint sheens (“Methods”) at concentrations ranging from ~1 to 40 g L^−1^. We observed no changes to the physical properties of the paint beyond a slight pink coloration that varied with concentration of copper–glass ceramic added. The bactericidal efficacy was found to depend on the paint formulation, the paint sheen, and the concentration of the copper–glass ceramic. In this paper, we focus on data at ~26 g L^−1^ of the copper–glass ceramic in an eggshell paint formulation as a typical use case for concentration and paint sheen (“Methods”). Plastic coupons coated with paint containing copper–glass ceramic particles were tested^11^ against four bacteria—the Gram-positive *Staphylococcus aureus* and the Gram-negative bacteria, *Pseudomonas aeruginosa*, *Klebsiella aerogenes*, and *Escherichia coli* (“Methods”). These bacteria are some of the leading causes of life-threatening hospital-acquired infections and can survive antibiotic intervention to proliferate resistant strains^29^. Greater than or equal to 99.9% reduction in colony counts of all four bacteria was observed within 2 h (Fig. 4, inset). *S. aureus* reduction kinetics show a steady decrease in colony counts over the course of 150 min, with EPA-prescribed 3-log kill achieved in ~1 h (Fig. 4); these results prove that the copper–glass ceramic has potent antibacterial properties.

To test the effectivity of the copper–glass ceramic against viruses, we tested the efficacy of coated coupons against murine norovirus (MNV-1), a test surrogate for human norovirus. Human norovirus is a non-enveloped virus, notorious for causing outbreaks of acute gastrointestinal illness aboard cruise ships. It is highly contagious and transmitted via contaminated surfaces, hand-to-hand contact, and ingesting contaminated food and water. We adapted an antiviral test protocol developed by Haldar et al.^30^ that is consistent with the principles of the EPA protocol for bactericidal efficacy (“Methods”). Complete inactivation of murine norovirus was observed on coated coupon surfaces (Supplementary Table 2).

Copper–glass ceramic was also evaluated as an in-can preservative of paint emulsions. Mainstream organic preservatives such as methylisothiazolinone are added to water-based emulsions to prevent spoilage by bacteria and fungi. Health-related risks associated with isothiazolinones^31^ have prompted tough regulatory measures and elicited an urgent search for safer alternatives. Using a standard test method for measuring the resistance of emulsion paints in a container to attack by microorganisms (ASTM D2574)^32^, we observed that the copper–glass ceramic was effective at preventing bacterial contamination by *P. aeruginosa* and *Enterobacter aerogenes (K. aerogenes)* (Supplementary Table 3) at only about 1/25th the concentration required for passing the EPA test against human pathogens.

### Toxicology and potential environmental impact

Animal toxicity studies of finely milled copper–glass ceramic particles revealed no significant concerns (Table 2; “Methods”; Supplementary Data 1; detailed reports available on request). We also evaluated the levels of leached copper from coatings to determine its potential environmental impact. The levels of Cu ions leached from paint films containing the copper–glass ceramic were studied over 5 days with higher than typical concentrations of the copper–glass ceramic (33 g L^−1^). The level of leached copper was ~550 ppb in the first 24 h, after which it dropped to a steady level of 20–35 ppb day^−1^ (Supplementary Table 4). These levels are well below the 1300 ppb Cu day^−1^ limit set by the EPA for drinking water.

**Table 2:** Toxicological profile of copper–glass ceramic powder in animal studies conducted in accordance with US EPA guidelines

Route of exposure	Study animal	Summary of results
Acute inhalation toxicity	Albino rat	LC_50_ >2.29 mg L^−1^
Acute dermal toxicity	Albino rat	LD_50_ >5050 mg kg^−1^
Acute oral toxicity	Albino rat	LD_50_ determined to be 5000 mg kg^−1^
Acute dermal irritation	Albino rat	Non-irritating to the skin
Acute eye irritation	Albino rabbit	Mildly irritating to the eyes
Skin sensitization	Guinea pig	Not a skin sensitizer

### Stability and longevity of antimicrobial potency

**Fig. 5:**
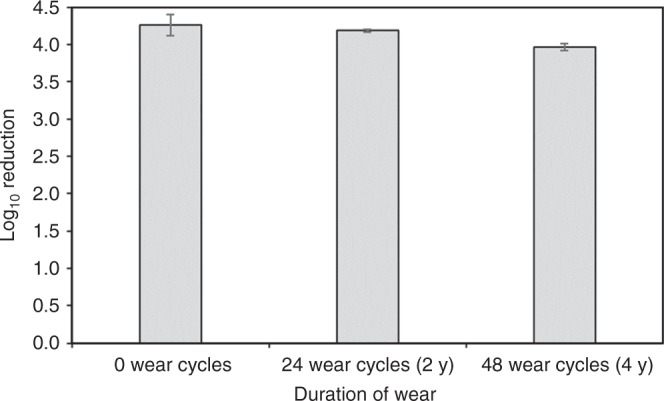
Efficacy of copper–glass ceramic particles after simulated wear. Plot of antimicrobial potency following washing and abrasion that illustrates longevity of copper–glass ceramic particles. Test paint coatings maintain bactericidal efficacy (>99.9%) even after 4 years of simulated wear (“Methods”). Each wear cycle represents 4× washing and abrasion every month. Log reduction calculations are differences in *S. aureus* colony counts between control and test coupons (both subjected to wear cycles). Data and error bars represent mean and standard deviation from *n* = 2 replicates

We explored the storage stability of the copper–glass ceramic and the ability of coatings containing the material to retain antimicrobial potency through simulated long-term use—considerations that are key to the practical deployment of the material. Accelerated aging studies of the copper–glass ceramic powder to simulate a 1-year period of storage showed no deterioration or corrosion (Supplementary Data 2). We evaluated the long-term potency of the liquid paint and the painted coupons containing the copper–glass ceramic; no decrease in potency was observed over a 6-month study period, suggesting conservation of antimicrobial potency as an additive in an aqueous emulsion and in the dry-film state. To simulate wear-and-tear and ability to withstand cleaning as prescribed by CDC guidelines for infection control, we subjected painted coupons with and without the copper–glass ceramic to rigorous washing and abrasion simulating up to 4 years of wear (“Methods”). Worn test coupons containing the copper–glass ceramic exhibited a >99.9% reduction in *S. aureus* colony counts (Fig. 5) relative to worn control coupons even after 48 cleaning cycles (192 passes of a wet scouring pad on the coating surface). We also evaluated the effect of repeated contamination/soiling of coupons with *S. aureus*. Control coupons showed an increase in bacterial bioburden following three repeat inoculations (Table 3). By contrast, test coupons demonstrated the ability to continuously reduce bacterial colony counts down to limit of detection. We made copper core–silica shell particles (as described in the literature^33^) as an alternative to a glass–ceramic host for Cu^1+^ ions. These nanoparticles were water dispersible and had an antimicrobial potency similar to the copper–glass ceramic but had to be stored under alcohol to preserve their orange-red color and antimicrobial properties.

**Table 3:** Continuous cleaning ability of copper–glass ceramic particles

No. of inoculations^a^	*S. aureus* colony counts after 2 h of exposure (CFU/coupon)	
	Control coupon	Test coupon
1	1.23E + 05	≤10
2	1.85E + 05	≤10
3	8.54E + 06	≤10

*CFU* colony-forming unit

^a^Each inoculation adds ~2E + 07 CFU/coupon (at 0, 2, and 4 h)

The high antimicrobial efficacy (≥99.9% microbial reduction in 2 h) of the copper–glass ceramic is achieved at very low surface concentrations of copper (~5%). An understanding of glass structure–property relationships and the steric constraints with H_3_O^+^ ion exchange led us to the copper–glass ceramic material with a fundamentally different mechanism for the release of copper ions. The work demonstrates and broadens the scope of rationally designed glasses as hosts for functional nanomaterials. The practical advantages of glass manufacturability contrast with the difficulties of scaling the bottoms up chemical syntheses of air-sensitive nanomaterials or host–guest complexes. Furthermore, the glass itself offers material properties and the potential for chemical functionalization that enable a broad range of applications. In an era of ever increasing concern with infectious diseases and frustration with the performance limitations and safety of organic biocides, the copper–glass ceramic described in this paper is particularly significant.

## Methods

### Preparation of glass and glass ceramic

The glass and glass ceramic materials were prepared from boalin sand (Coarse Berkely Fine Special; US Silica), calcined alumina (A2, Unground Classified; Almatis), cupric oxide (325 mesh; Ceramic Color and Chemical), aluminum metaphosphate (Powder, Technical Grade; Chemical Distributors Inc.), technical grade boric acid (Optibor TG; Chemical Distributors Inc.), and potassium carbonate (Food additive grade; Amrex Chemical) raw materials. The raw materials were mixed in a Turbula mixer, then melted for 6 h at 1600 °C in covered fused quartz crucibles. The molten glasses were then poured from the crucibles onto a clean stainless-steel table and transferred to annealing furnaces set to the previously determined anneal point for each glass. Glasses were heat treated at the anneal point temperature for 6 h and then cooled to room temperature at a cooling rate of 100° C h^−1^.

### Milling of copper–glass ceramic

The glass samples were hammered into small chunks, magnetically cleaned, and fed into a ceramic disc pulverizer to produce a jet milling feedstock size to pass through a 20-mesh sieve (850-µm openings). The glass grains were processed on a ceramic lined laboratory size 2” Sturtevant Micronizer® air jet mill and reduced to a fine micron size powder with a narrow particle distribution. The jet milled powder was then screened at 325 mesh (45-µm openings) to remove any foreign debris and unground particles.

### Coupon preparation

Finely milled copper–glass ceramic particles were weighed and mixed with commercial paints (PPG Olympic One, Eggshell Interior Paint, PPG Diamond Interior Paint +Primer) using an overhead stirrer (Fisher Scientific) for 3–5 min to prepare test paint containing 26 g L^−1^ of copper–glass ceramic particles. The emulsion was stirred again for 3–5 min prior to coupon preparation. 1” × 1” test coupons (BYK-Gardner 5015 byko-chart black scrub test panels P121-10N) were painted with primer (Zinsser® Bulls Eye 1-2-3 Water-Base Primer; 3 mils) and allowed to dry overnight at ambient temperature and humidity. Two coats of paint (3 mils each) containing copper–glass ceramic were applied sequentially and each layer was allowed to dry for ≥24 h. Untreated control coupons were similarly prepared using paints without copper–glass ceramic particles. On the day of testing, coupons were exposed to ultraviolet light (Thermo Scientific 13—Series Class II, Type A2 Biological Safety Cabinet; wavelength 254 nm) on both sides for approximately 15 min. Stainless-steel coupons, used as reference surfaces, were cleaned and sterilized by immersion in a 75% ethanol solution followed by rinsing with deionized (DI) water.

### Microorganisms and cell lines

Bacterial strains used were *S. aureus* (ATCC 6538), *K. aerogenes* (ATCC 13048), *P. aeruginosa* (ATCC 15442), and *E. coli* (ATCC 35150). Vials containing each bacterial stock culture were stored at −80 °C until use.

Virucidal efficacy tests were conducted at Accuratus Lab Services, MN. Accuratus used murine norovirus, strain S99, obtained from Friedrich-Loeffler-Institut, Greifswald, Germany and conducted infectivity assays using RAW 264.7 cells, a continuous mouse macrophage cell line.

### Bactericidal efficacy tests

Bactericidal efficacy tests including study controls were performed as described in the EPA test for efficacy of copper alloy surfaces as a sanitizer^11^. Each coupon was tested in triplicate.

Twenty-µL aliquots of thawed bacterial cultures were added to 10 mL Tryptic Soy Broth (Teknova). These bacterial suspensions were serially incubated 3× at 36 °C (*K. aerogenes* at 30 °C) for 18–24 h in an orbital shaker (New Brunswick Scientific) and then 1× in polypropylene snap tubes (Fisher Healthcare) for 48 h. Cultures were subsequently mixed on a vortex mixer (VWR Scientific) and allowed to settle. The upper two thirds of suspension from each tube was aspirated and OD_600_ measured (Smart Spec Spectrophotometer 3000, Bio-Rad) for bacterial density estimation. The culture was diluted with phosphate buffer saline (Gibco Life Technologies) to achieve a bacterial inoculum concentration near a target value of 1.0 × 10^7^ colony-forming units (CFU) mL^−1^. Organic soil load containing 0.25 mL of 5% fetal bovine serum (Gibco Life Technologies) and 0.05 mL Triton X-100 (Amresco Pro Pure) was added to 4.70 mL bacterial suspension to aid in spreading the inoculum.

Each coupon was inoculated with 20 µL of the bacterial test culture. The inoculum volume was spread evenly using bent sterile pipette tips (Mettler-Toledo) to ensure full and even coverage, spreading as close to the edge of the coupon as possible. Coupons were then incubated in a controlled environment set at 42% relative humidity (RH) and 23 °C for a period of 120 min.

Following the 120-min exposure period, coupons were neutralized in Letheen broth (Gen Lab). Ten-fold serial dilutions of the neutralized solutions were plated using standard spread plate technique on Tryptic Soy Agar plates and incubated for 48 h at 36 °C (30 °C for *K. aerogenes*) to yield countable numbers of survivors (approximately 20–200 colonies per plate).

### Simulated wear of copper–glass ceramic particles in coatings

Paint panels containing copper–glass ceramic (26 g L^−1^) were subjected to wear procedure (washing and abrasion), simulating a worst-case scenario of 4× cleaning every month for up to 4 years.

Simulated wear was performed using an Elcometer 5750 Taber® Linear Abraser. Cleaning solution was prepared with a general multipurpose detergent (Best Yet Citrus Cleaner™) at prescribed dilution in DI water (1:64). A scouring pad (3 M ScotchBrite® Light Duty Cleaning Pad (1” × 1”)) was saturated with cleaning solution—2 sprays applied from a distance of 2–3 ”, using a trigger spray bottle (Great Value™). The pad was attached to an aluminum block attachment (ScotchBrite® Kit, Taber Industries) using double-sided adhesive tape. No additional accessory weights were added to the spline-shaft of the linear abraser (base load of 350 g) to keep the pressure similar to that applied in standard paint washability tests such as ASTM D3450. Speed was set to 30 cycles min^−1^, cycle counter at 2, and stroke length was set to 4”. A 1” × 4” painted panel was secured below the ScotchBrite® pad and 2 cycles were run, representing 1 complete wear cycle (4 passes of the wet scouring pad against the surface). The panel was dried for ≥10 min before the next wear cycle was performed. This process was repeated for 24 cycles (96 passes of the wet scouring pad) to simulate wear and washing for up to 2 years and 48 cycles (192 passes of the wet scouring pad) to simulate cleaning for up to 4 years. After completion of wear cycles, panels were cut into 1” × 1” coupons and tested for bactericidal efficacy against *S. aureus* (ATCC 6538). Bacterial colony counts on worn test coupons were subtracted from colony counts on similarly worn control coupons to measure reductions.

### Continuous cleaning ability of copper–glass ceramic

Three sets of test coupons (containing copper–glass ceramic) and 3 sets of control coupons were used. All coupons were inoculated with 20 µL of bacterial inoculum (*t* = 0). Following 2 h of incubation at 23 °C and 42% RH, one test and one control coupon were neutralized and removed for quantitative recovery. The remaining coupons were reinoculated a second time (*t* = 2 h) with 20 µL of culture using a calibrated pipette. Another test and control coupon were removed for quantitative recovery and the remaining set reinoculated a third time (*t* = 4 h). This set of coupons was neutralized and enumerated at *t* = 6 h. At each recovery time (2, 4, and 6 h), control and test coupons were neutralized and enumerated.

### Virucidal efficacy tests

Protocol for antiviral efficacy was adapted from Haldar et al.^30^ and conducted at Accuratus Lab Services, MN.

Coupons were inoculated with 20 µL murine norovirus using the same procedure used for bacteria and incubated in a controlled chamber set to RH of 50% and temperature of 20 °C. After 120 min, 1.0 mL aliquots of 2× Minimum Essential Medium (Gibco Life Technologies) were pipetted individually onto each test and control coupon. The surface of each coupon was scraped with sterile plastic cell scrapers (Bioscience) and the test medium collected and passed through Sephadex columns (GE Healthcare). The filtrates were then serially diluted ten-fold and assayed for infectivity in RAW 264.7 cells.

### Calculations of log and percentage of reductions

Log and percentage of reductions for bactericidal efficacy tests measure differences in CFUs between control and test coupons^11^. For virucidal efficacy tests, PFU_50_ signifies the concentration at which 50% of the RAW 264.7 cells are infected when the well plate upon which cells have been cultured is inoculated with a diluted solution of viral fluid from either test or control coupons. Log and percentage of reductions measure differences in PFU_50_ between control and test coupons.

### Acute toxicity testing in animals

Acute toxicity testing was conducted by Stillmeadow Inc, TX. Methods and results are summarized in Supplementary Data 1.

### Molecular dynamics simulations

Molecular dynamics simulations were carried out on a cubic sample made up of approximately 2000 atoms with random coordinates equilibrated at 4000 K for 1 ns. The high temperature melt was quenched to 300 K continuously over a period of 8 ns in an NPT (constant number of atoms, constant pressure, and constant temperature) essemble under ambient pressure^34^. Finally, the glass was relaxed at 300 K under atmospheric pressure for 1 ns. Periodic boundary conditions were applied in all directions. The widely used force field for oxides developed by Pedone et al.^35^ was used. The visualization of the local atomic structure was facilitated by Ovito^36^.

### Reporting summary

Further information on research design is available in the Nature Research Reporting Summary linked to this article.

## Supplementary information

Supplementary Information

Reporting Summary

## Data Availability

Data generated during and/or analyzed during the current study are included in this published article (and its Supplementary Information file). Detailed reports that support the findings in this study are available from the corresponding author upon reasonable request.
