# Supplementary material for: Copper-containing glass ceramic with high antimicrobial efficacy
Source: Nat Commun. 2019 Apr 30;10:1979. doi: 10.1038/s41467-019-09946-9 (PMC6491652; doi:10.1038/s41467-019-09946-9)
Supplement: Supplementary file 1 — Supplementary Information [file 41467_2019_9946_MOESM1_ESM.pdf]

Supplementary Information

Copper-containing glass ceramic with high antimicrobial efficacy

Supplementary Table 1 Composition of leachate from copper-glass ceramic particles

Analyzed Composition (ppb)	Copper Glass-Ceramic*	Water (ref)
**Al**	18	4
**B**	2670	20
**Ca**	26	72
**Cu**	486	4
**K**	22300	20
**Mg**	7	11
**Na**	136	100
**P**	5450	50
**Si**	2710	20
**W**	10	10
*Supernatant from a concentration of 26g/L copper-glass ceramic in DI water, leached for 4 hours		

Supplementary Table 2: Reduction in Murine Norovirus following 2-hour exposure to paint containing copper-glass ceramic particles

**Murine Norovirus***	**Replicate #1**	**Replicate #2**	**Replicate #3**	**Mean**
Control Coating (PFU_{50}/250µL)	$10^{5.00}$	$10^{4.75}$	$10^{5.50}$	$10^{5.08}$
Coating + copper-glass ceramic particles (PFU_{50}/250µL)	$\leq 10^{1.50}$	$\leq 10^{1.50}$	$\leq 10^{1.50}$	$\leq 10^{1.50}$
**Mean Percent Reduction**	**≥99.97%**			
**Mean Log_{10} Reduction**	**≥3.58 Log_{10}**			

*PFU50 signifies the concentration at which 50% of the cells are infected when a test tube or well plate upon which cells have been cultured is inoculated with a diluted solution of viral fluid.

Supplementary Table 3: 'In-Can' preservative performance of Copper-Glass Ceramic particles

***Pseudomonas aeruginosa* (ATCC 10145)**	**Number of weeks (challenge once a week)**			
	**1**	**2**	**3**	**4**
Control coating	1	3	4	4
Coating + copper-glass ceramic particles	0	0	0	0
***Enterobacter aerogenes* (ATCC 13048)**	**Number of weeks (challenge once a week)**			
	**1**	**2**	**3**	**4**
Control coating	1	1	2	4
Coating + copper-glass ceramic particles	0	0	0	0

0 = No bacterial recovery
1= Trace of contamination (1 to 9 colonies)
2 = Light contamination (10 to 99 colonies)
3 = Moderate contamination (< 100 distinct colonies)
4 = Heavy contamination (continuous smear of growth)

Supplementary Table 4: Leached copper from coatings containing copper-glass ceramic particles

Day	Cu (ppb)*	Average Cu (ppb)*
**1**	500	550
	710	
	440	
**2**	32	35
	40	
	33	
**3**	28	20
	16	
	15	
**5**	28	24
	29	
	14	

*Supernatant from paint-films containing copper-glass ceramic at a concentration of 26g/L, leached for 24 hours in DI water

Supplementary Data 1: Summary of acute toxicology studies

All acute toxicity studies were performed by Stillmeadow Incorporated, TX per EPA test guidelines in compliance with Good Laboratory Practices.

Acute oral toxicity in rats (Test Guideline OCSPP 870.1100)

Copper-glass ceramic particles were evaluated for acute oral toxicity potential in female albino rats when administered as a gavage dose at 5000 mg/kg. Since the test substance failed the limit test, a main test was conducted following up-and-down procedure (UDP) at 175, 550, 1750 and 5000 mg/kg. The study was terminated following stopping rules of this procedure. Mortality occurred only at the 5000 mg/kg level; one animal exhibited severe symptoms and weight loss by Day 14 study termination. Clinical signs included activity decrease, blue feces/urine, decreased defecation, distended abdomen, cyanosis, hunched posture, hypothermia, emaciation, ocular discharge and piloerection. Survivors had signs on days 0, and 6-10. Animals surviving to termination exhibited weekly weight gain during the study. Abnormal necropsy findings occurred only in the animals dying during the test, and pertained to facial/anogenital areas, lungs, liver, spleen, and contents of the gastrointestinal tract. The test substance acute oral $LD_{50,}$ indicated by the data, was estimated to be 5000 mg/kg.

Acute dermal toxicity in rats (Test guideline OCSPP 870.1200)

Copper-glass ceramic particles were evaluated for dermal toxicity potential and relative skin irritancy when a single dose at 5050 mg/kg, moistened with deionized (DI) water, was applied to the intact skin of albino rats. No mortality occurred during the study. There were no clinical

signs of toxicity or signs of dermal irritation at any time throughout the study. Animals exhibited weekly weight gain during the study. Gross necropsy conducted at study termination revealed no observable abnormalities. The test substance LD_{50} was determined to be greater than 5050 mg/kg.

Acute eye irritation in rabbits (Test guideline OCSPP 870.2400)

Acute eye irritation study was conducted on three albino rabbits (1 to start, 2 after) using test substance (copper-glass ceramic particles). 100 mg of test substance was placed in the conjunctival sac of the right eye of the animal selected for testing. Any treated eyes were washed with room temperature deionized (DI) water for one minute after recording the 2-hour observation.

The number of animals testing "positive" for each parameter vs. number of animals observed is presented as follows:

	**Time After Treatment**				
	**Hours**				**Day**
	**1**	**24**	**48**	**72**	**4**
Cornea Opacity	1/3	0/3	0/3	0/3	0/1
Iritis	0/3	0/3	0/3	0/3	0/1
Conjunctivae					
Redness	2/3	2/3	2/3	0/3	0/1
Chemosis	1/3	0/3	0/3	0/3	0/1

There were no positive effects exhibited in any eye at 72 hours after treatment. Therefore, the test substance is assigned Toxicity Category III. The test substance is mildly irritating.

Acute dermal irritation in rabbits (Test guideline OCSPP 870.2500)

A primary dermal irritation study was conducted on three albino rabbits. Each test site (one intact site per animal) was treated with 500 mg of copper-glass ceramic particles moistened with DI water and covered with semi-permeable dressing. Particles were kept in contact with animal skin for 4 hours. Observations for dermal irritation and defects were made at 1, 24, 48 and 72 hours after unwrapping the dressing.

Irritation scores derived from respective erythema and edema scores through 72-hour observations are tabulated below:

	Erythema				Edema				
	Hours after Unwrapping				Hours after unwrapping				
	1	24	48	72	1	24	48	72	Irritation Scores
Rabbit 1	0	0	0	0	0	0	0	0	0
Rabbit 2	0	0	0	0	0	0	0	0	0
Rabbit 3	0	0	0	0	0	0	0	0	0
Primary Irritation Index (PII) = 0									

Copper-glass ceramic particles are rated non-irritating based on a PII = 0. Based on scores at 72-hours, copper-glass ceramic particles are assigned Toxicity Category IV.

Skin sensitization in guinea pigs (Test guideline OCSPP 870.2600)

Skin sensitization study was conducted on 15 male and 15 female short-haired albino guinea pigs. Animals were assigned to one of two groups, designed naive control (5/gender) and test (10/gender). Naive control animals remained untreated during the induction phase of the study. Animals in the test group were treated with 400 mg of test substance moistened with DI water

(selected from range-finding). Test animals were treated once weekly for three weeks, for a total of three inductions. After a two-week rest period, all animals (both groups) were challenged at a virgin test site with an application of 400 mg of copper-glass ceramic moistened with DI water. Copper-glass ceramic particles produced no reaction in either test animals or naïve control animals after the challenge treatment and is not a sensitizer in guinea pigs.

Acute inhalation toxicity (Test guideline OCSPP 870.1300)

Five male and five female albino rates were exposed for four hours to an aerosol generated from copper-glass ceramic particles at 2.29 mg/L. No mortality occurred during the study. Clinical signs include decrease in activity, piloerection and test substance on muzzle, no longer evident by Day 1. Animals exhibited weekly weight gain with the exception of one test animal that lost weight between Days 0 and 7. Gross necropsy revealed no observable abnormalities except discolored lungs. Acute inhalation LC_{50} is greater than 2.29 mg/L.

Supplementary Data 2: Accelerated storage stability of copper-glass ceramic particles (OPPTS 830.6317)

Storage stability of copper-glass ceramic particles was studied at Still Meadows Lab, TX. On day 0, samples of copper-glass ceramic were analyzed by a validated titration method and were found to contain an average of 32.85% copper oxide. Titration was conducted again on samples incubated at 54°C for 14 days, which simulates storage over a 1-year period. Average copper oxide concentration was found to be 32.54% demonstrating analytical stability of copper-glass ceramic.
